# Supplementary material for: Azole-induced cell wall carbohydrate patches kill Aspergillus fumigatus
Source: Nat Commun. 2018 Aug 6;9:3098. doi: 10.1038/s41467-018-05497-7 (PMC6078979; doi:10.1038/s41467-018-05497-7)
Supplement: Supplementary file 1 — Supplementary Information [file 41467_2018_5497_MOESM1_ESM.pdf]

Azole-induced cell wall carbohydrate patches kill *Aspergillus fumigatus*.

Supplementary Information

Geißel et al.

Supplementary figure 1

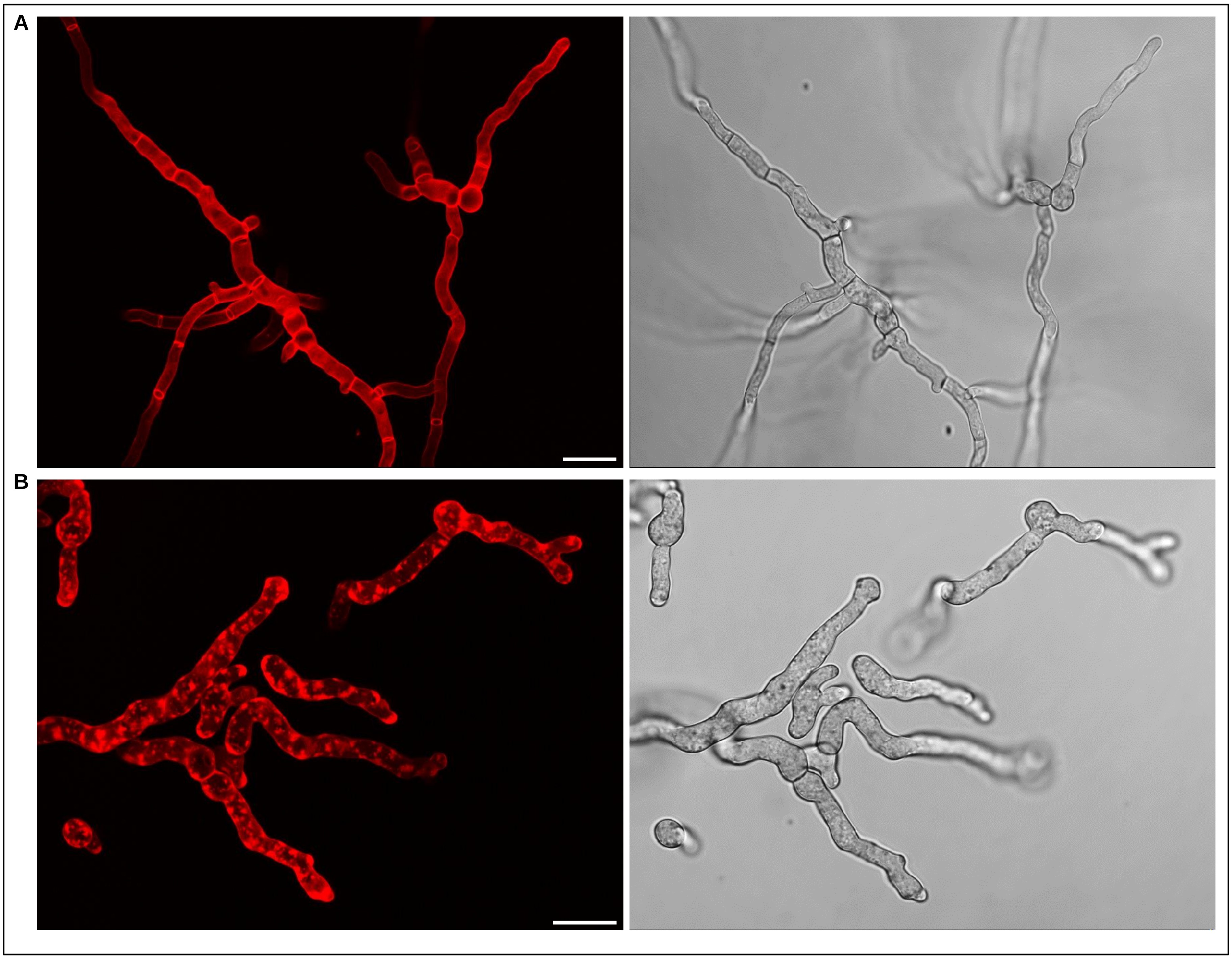

Supplementary Figure 1. Exposure of *A. fumigatus* hyphae to voriconazole triggers the formation of patch-like structures that are strongly stained by trypan blue. (A and B) Conidia of *A. fumigatus* wild type were inoculated in Sabouraud medium and incubated at 37 °C. (B) After 10 h incubation medium was supplemented with 1.27 µg ml^{-1} voriconazole. (A and B) After a total of approximately 15 h incubation, hyphae were stained with trypan blue and analyzed with a confocal laser scanning microscope. Depicted are representative images of optical stacks of trypan blue fluorescence (red, left panels) that cover the entire hyphae in focus, or bright field images (right panels). Bars represent 20 µm.

Supplementary figure 2

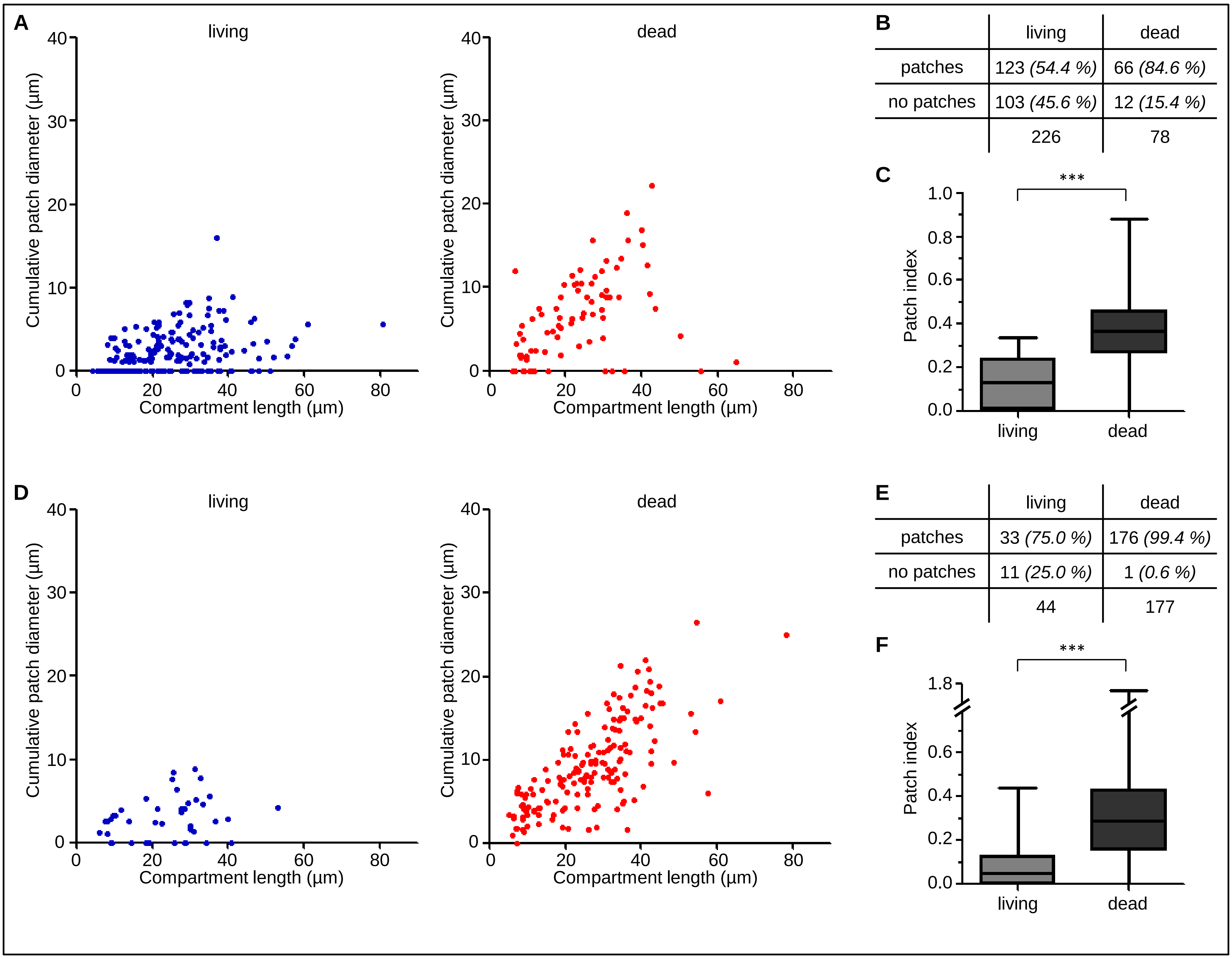

B

	living	dead
patches	123 *(54.4 %)*	66 *(84.6 %)*
no patches	103 *(45.6 %)*	12 *(15.4 %)*
	226	78

E

	living	dead
patches	33 *(75.0 %)*	176 *(99.4 %)*
no patches	11 *(25.0 %)*	1 *(0.6 %)*
	44	177

Supplementary Figure 2. The presence and size of the cell wall carbohydrate patch correlate with death of individual hyphae. (A – F) Conidia of the *cycA*$_{tetOn}$ mutant expressing mitochondria-targeted GFP were inoculated in Sabouraud medium under repressed conditions. After 10 h of incubation at 37 °C, medium was supplemented with 2.4 µg ml^{-1} (A – C) or 3.2 µg ml^{-1} (D – F) voriconazole and incubated for another 15 h. Hyphae were stained with calcofluor white, and analyzed with time-lapse laser scanning microscopy. Short video sequences of multiple hyphae were taken and each hyphal compartment was analyzed for viability, compartment length and cumulative diameter of the containing cell wall carbohydrate patches. The depicted results are based on video microscopic data obtained in two independent experiments (2.4 or 3.2 µg ml^{-1} voriconazole, respectively) from three technical replicates. 304 (A – C) or 221 (D – F) hyphal compartments were analyzed in total. Very similar results were obtained in an independent experiment with the *rip1*$_{tetOn}$ strain under similar conditions (Figure 7). (A and D) The graphs indicate the cumulative patch diameter and compartment length for each living (blue) and dead (red) compartment. (B and E) Absolute and relative numbers for living and dead compartments that exhibit patches or no patches. A significant number of living compartments exhibit no patches, while most (B; less fungicidal azole concentration) or almost all (E; fungicidal azole concentration) dead compartments have patches. (C and F) Dead compartments exhibit a significantly higher ratio of the cumulative patch diameter and compartment length (patch index). Statistical significance (***, $p \leq 0.001$) was calculated with a Mann-Whitney test.
