# Supplementary material for: Azole-induced cell wall carbohydrate patches kill Aspergillus fumigatus
Source: Nat Commun. 2018 Aug 6;9:3098. doi: 10.1038/s41467-018-05497-7 (PMC6078979; doi:10.1038/s41467-018-05497-7)
Supplement: Supplementary file 3 — Description of Additional Supplementary Files [file 41467_2018_5497_MOESM3_ESM.pdf]

Description of Additional Supplementary Files

File Name: Supplementary Movie 1

Description: **Manifestations of voriconazole-induced death: bursting hyphae (GFP signal).** *A. fumigatus* wild type conidia expressing mitochondria-targeted GFP were inoculated in Sabouraud medium in a live cell microscopy slide and incubated at 37 °C. After 9 h, medium was supplemented with 0.4 µg ml^{-1} voriconazole. The fate of the hyphae in focus was subsequently followed over time with confocal laser scanning microscopy. The movie starts approximately 15 minutes after addition of the azole and shows the GFP signal of optical stacks covering the entire hyphae in focus. An overlay of bright field and the GFP signal is also available as separate movie file (Supplementary Movie 2).

File Name: Supplementary Movie 2

Description: **Manifestations of voriconazole-induced death: bursting hyphae (overlay of GFP signal and bright field).** *A. fumigatus* wild type conidia expressing mitochondria-targeted GFP were inoculated in Sabouraud medium in a live cell microscopy slide and incubated at 37 °C. After 9 h, medium was supplemented with 0.4 µg ml^{-1} voriconazole. The fate of the hyphae in focus was subsequently followed over time with confocal laser scanning microscopy. The movie starts approximately 15 minutes after addition of the azole and shows an overlay of bright field and the GFP signal of optical stacks covering the entire hyphae in focus. The GFP signal without bright field is also available as separate movie file (Supplementary Movie 1).

File Name: Supplementary Movie 3

Description: **Manifestations of voriconazole-induced death: mitochondrial fragmentation (GFP signal).** *A. fumigatus* wild type conidia expressing mitochondria-targeted GFP were inoculated in Sabouraud medium in a live cell microscopy slide and incubated at 37 °C. After 9 h, medium was supplemented with 1.27 µg ml^{-1} voriconazole. The of fate the hypha in focus was subsequently followed over time with confocal laser scanning microscopy. The movie starts approximately 15 minutes after addition of the azole and shows the GFP signal of optical stacks covering the entire hypha in focus. An overlay of bright field and the GFP signal is also available as separate movie file (Supplementary Movie 4).

File Name: Supplementary Movie 4

Description: **Manifestations of voriconazole-induced death: mitochondrial fragmentation (overlay of GFP signal and bright field).** *A. fumigatus* wild type conidia expressing mitochondria-targeted GFP were inoculated in Sabouraud medium in a live cell microscopy slide and incubated at 37 °C. After 9 h, medium was supplemented with 1.27 µg ml^{-1} voriconazole. The of fate the hypha in focus was subsequently followed over time with confocal laser scanning microscopy. The movie starts approximately 15 minutes after addition of the azole and shows an overlay of bright field and the GFP signal of optical stacks covering the entire hypha in focus. The GFP signal without bright field is also available as separate movie file (Supplementary Movie 3).

File Name: Supplementary Movie 5

Description: **Manifestations of voriconazole-induced death: lysis of mitochondria (GFP signal).** *A. fumigatus* wild type conidia expressing mitochondria-targeted GFP were inoculated in Sabouraud medium in a live cell microscopy slide and incubated at 37 °C. After 9 h, medium was supplemented with 1.27 µg ml^{-1} voriconazole. The of fate the hyphae in focus was subsequently followed over time with confocal laser scanning microscopy. The movie starts approximately 15 minutes after addition of the azole and shows the GFP signal

of optical stacks covering the entire hyphae in focus. An overlay of bright field and the GFP signal is also available as separate movie file (Supplementary Movie 6).

File Name: Supplementary Movie 6
Description: **Manifestations of voriconazole-induced death: lysis of mitochondria (overlay of GFP signal and bright field).** *A. fumigatus* wild type conidia expressing mitochondria-targeted GFP were inoculated in Sabouraud medium in a live cell microscopy slide and incubated at 37 °C. After 9 h, medium was supplemented with 1.27 µg ml^{-1} voriconazole. The of fate the hyphae in focus was subsequently followed over time with confocal laser scanning microscopy. The movie starts approximately 15 minutes after addition of the azole and shows an overlay of bright field and the GFP signal of optical stacks covering the entire hyphae in focus. The GFP signal without bright field is also available as separate movie file (Supplementary Movie 5).

File Name: Supplementary Movie 7
Description: **Exemplary movie of the quantitative analysis of voriconazole-induced death manifestations (0.4 µg ml^{-1} voriconazole; GFP signal).** Wild type conidia expressing mitochondria-targeted GFP were inoculated in Sabouraud medium in a live cell microscopy slide and incubated at 37 °C. After 9 h, medium was supplemented with 0.4 µg ml^{-1} voriconazole. The fate of individual hyphae in focus was subsequently followed over time with confocal laser scanning microscopy. The movie starts approximately 15 minutes after addition of the azole and shows the GFP signal of optical stacks covering the entire hyphae in focus. An overlay of bright field and the GFP signal is also available as separate movie file (Supplementary Movie 8).

File Name: Supplementary Movie 8
Description: **Exemplary movie of the quantitative analysis of voriconazole-induced death manifestations (0.4 µg ml^{-1} voriconazole; overlay of GFP signal and bright field).** Wild type conidia expressing mitochondria-targeted GFP were inoculated in Sabouraud medium in a live cell microscopy slide and incubated at 37 °C. After 9 h, medium was supplemented with 0.4 µg ml^{-1} voriconazole. The fate of individual hyphae in focus was subsequently followed over time with confocal laser scanning microscopy. The movie starts approximately 15 minutes after addition of the azole and shows an overlay of bright field and the GFP signal of optical stacks covering the entire hyphae in focus. The GFP signal without bright field is also available as separate movie file (Supplementary Movie 7).

File Name: Supplementary Movie 9
Description: **Exemplary movie of the quantitative analysis of voriconazole-induced death manifestations (1.27 µg ml^{-1} voriconazole; GFP signal).** Wild type conidia expressing mitochondria-targeted GFP were inoculated in Sabouraud medium in a live cell microscopy slide and incubated at 37 °C. After 9 h, medium was supplemented with 1.27 µg ml^{-1} voriconazole. The fate of individual hyphae in focus was subsequently followed over time with confocal laser scanning microscopy. The movie starts approximately 15 minutes after addition of the azole and shows the GFP signal of optical stacks covering the entire hyphae in focus. An overlay of bright field and the GFP signal is also available as separate movie file (Supplementary Movie 10).

File Name: Supplementary Movie 10
Description: **Exemplary movie of the quantitative analysis of voriconazole-induced death manifestations (1.27 µg ml^{-1} voriconazole; overlay of GFP signal and bright field).** Wild type conidia expressing mitochondria-targeted GFP were inoculated in Sabouraud medium in a live cell microscopy slide and incubated at 37 °C. After 9 h, medium

was supplemented with 1.27 µg ml^{-1} voriconazole. The fate of individual hyphae in focus was subsequently followed over time with confocal laser scanning microscopy. The movie starts approximately 15 minutes after addition of the azole and shows an overlay of bright field and the GFP signal of optical stacks covering the entire hyphae in focus. The GFP signal without bright field is also available as separate movie file (Supplementary Movie 9).

File Name: Supplementary Movie 11
Description: **Azole-induced plasma membrane invaginations (bright field).** *A. fumigatus* conidia expressing GFP-tagged membrane-anchored Wsc1 were inoculated in Sabouraud medium in a live cell microscopy slide. After 9 h incubation at 37 °C, medium was supplemented with 0.53 µg ml^{-1} voriconazole. The fate of the hyphae in focus was subsequently followed over time with confocal laser scanning microscopy. The movie starts approximately 15 minutes after addition of the azole and shows bright field. The GFP signal and an overlay of the GFP signal and bright field are also available as separate movie files (Supplementary Movie 12 and 13).

File Name: Supplementary Movie 12
Description: **Azole-induced plasma membrane invaginations (GFP signal).** *A. fumigatus* conidia expressing GFP-tagged membrane-anchored Wsc1 were inoculated in Sabouraud medium in a live cell microscopy slide. After 9 h incubation at 37 °C, medium was supplemented with 0.53 µg ml^{-1} voriconazole. The fate of the hyphae in focus was subsequently followed over time with confocal laser scanning microscopy. The movie starts approximately 15 minutes after addition of the azole and shows the GFP signal of a single fluorescence cross section (glow dark color scheme). Bright field and an overlay of bright field and the GFP signal are also available as separate movie files (Supplementary Movie 11 and 13).

File Name: Supplementary Movie 13
Description: **Azole-induced plasma membrane invaginations (overlay of GFP signal and bright field).** *A. fumigatus* conidia expressing GFP-tagged membrane-anchored Wsc1 were inoculated in Sabouraud medium in a live cell microscopy slide. After 9 h incubation at 37 °C, medium was supplemented with 0.53 µg ml^{-1} voriconazole. The fate of the hyphae in focus was subsequently followed over time with confocal laser scanning microscopy. The movie starts approximately 15 minutes after addition of the azole and shows an overlay of bright field and the GFP signal of a single fluorescence cross section (glow dark color scheme). Bright field and the GFP signal without bright field are also available as separate movie files (Supplementary Movie 11 and 12).

File Name: Supplementary Movie 14
Description: **Exemplary movie of calcofluor white-stained dead and living hyphae of the conditional complex III mutant after exposure to partially fungicidal azole concentrations (bright field).** Conidia of the conditional complex III mutant ($rip1_{tetOn}$) expressing mitochondria-targeted GFP were inoculated in Sabouraud medium under repressed conditions. After 10 h of incubation at 37 °C, medium was supplemented with 2.4 µg ml^{-1} voriconazole and incubated for another 15 h. Hyphae were stained with calcofluor white, and analyzed with time-lapse laser scanning microscopy. Short movies (GFP signal, calcofluor white signal, bright field) of multiple hyphal compartments were taken and analyzed for viability, compartment length and cumulative diameter of the containing cell wall carbohydrate patches. The movie shows bright field. An overlay of the calcofluor white and the GFP signal is also available as separate movie file (Supplementary Movie 15).

File Name: Supplementary Movie 15

Description: **Exemplary movie of calcofluor white-stained dead and living hyphae of the conditional complex III mutant after exposure to partially fungicidal azole concentrations (overlay of GFP and calcofluor white signal).** Conidia of the conditional complex III mutant ($\textit{rip1}_{tetOn}$) expressing mitochondria-targeted GFP were inoculated in Sabouraud medium under repressed conditions. After 10 h of incubation at 37 °C, medium was supplemented with 2.4 µg ml^{-1} voriconazole and incubated for another 15 h. Hyphae were stained with calcofluor white, and analyzed with time-lapse laser scanning microscopy. Short movies (GFP signal, calcofluor white signal, bright field) of multiple hyphal compartments were taken and analyzed for viability, compartment length and cumulative diameter of the containing cell wall carbohydrate patches. The movie shows an overlay of the calcofluor white and the GFP signal of optical stacks covering the entire hyphae in focus. Bright field is also available as separate movie file (Supplementary Movie 14).
